# Textile-Based Sensors for Biosignal Detection and Monitoring

**DOI:** 10.3390/s21186042

**Published:** 2021-09-09

**Authors:** Tomasz Blachowicz, Guido Ehrmann, Andrea Ehrmann

**Affiliations:** 1 Center for Science and Education, Institute of Physics, Silesian University of Technology, 44-100 Gliwice, Poland; tomasz.blachowicz@polsl.pl; 2 Virtual Institute of Applied Research on Advanced Materials (VIARAM); guido.ehrmann@gmx.de; 3 Faculty of Engineering and Mathematics, Bielefeld University of Applied Sciences, 33619 Bielefeld, Germany

**Keywords:** ECG, EMG, sweat, health condition, health status, elderly, firefighters, sportsman

## Abstract

Biosignals often have to be detected in sports or for medical reasons. Typical biosignals are pulse and ECG (electrocardiogram), breathing, blood pressure, skin temperature, oxygen saturation, bioimpedance, etc. Typically, scientists attempt to measure these biosignals noninvasively, i.e., with electrodes or other sensors, detecting electric signals, measuring optical or chemical information. While short-time measurements or monitoring of patients in a hospital can be performed by systems based on common rigid electrodes, usually containing a large amount of wiring, long-term measurements on mobile patients or athletes necessitate other equipment. Here, textile-based sensors and textile-integrated data connections are preferred to avoid skin irritations and other unnecessary limitations of the monitored person. In this review, we give an overview of recent progress in textile-based electrodes for electrical measurements and new developments in textile-based chemical and other sensors for detection and monitoring of biosignals.

## 1. Introduction

Transforming textiles from objects protecting people from temperature, rain, etc., into functional textiles with additional properties belongs to the emerging trends of our time. These so-called smart textiles often contain electronics, integrated by different degrees, to create special designs; to couple jackets to smartphones; to enable tracking of firefighters or automatic emergency calls for avalanche victims; or to allow for the detection of biosignals, especially of athletes, the elderly and ill people who should be monitored for longer durations [1,2,3,4,5,6]. Besides these sensors, it is necessary to include communication, a power source and a data processor, again with different degrees of integration [7,8,9].

While full integration of sensors and the additional electronics into textile fabrics is not always easy at the recent state of technology, the advantages of this approach are clear. Electrodes with direct skin contact can be prepared from more skin-friendly material and in more comfortable shapes than, for example, common ECG electrodes or the relatively rigid chest straps known from pulse measurements in sports. The additional possibility to embed all necessary cables into the textiles, too, makes a long-term ECG based on textile electrodes and textile-integrated electronics much more comfortable than the still common version with rigid equipment, several cables and glued electrodes [10,11,12].

While the first textile-based sensors used conductive yarns or fabrics to measure electronic signals, such as voltage or conductivity to measure ECG or strain, more sophisticated material compositions have been investigated in the meantime, allowing for a broader spectrum of measurements. Here, we give an overview of recent research on diverse physical sensors, usually based on detecting electronic properties, and some chemical sensors which can be used to detect different gases, but also diverse health-related biomarker molecules [13,14,15], which can be created by combining conductive, semi-conductive and isolating materials with different dielectric properties by different techniques on the fiber or yarn level or on complete textile fabrics. This updated overview furthermore discusses the advantages and still existing challenges of textile-based biosensors, focusing on most recent, as well as fundamental, papers to cover a broad range of possibilities and problems in this field of research.

## 2. ECG Measurement

Measuring ECG signals belongs to the most common applications of textile electrodes. Such electrodes can be produced from conductive yarns by weaving, knitting, felting, moss embroidery, etc., or they can be prepared by conductive coatings on isolating or conductive yarns [16,17]. The main problem which has to be solved is the skin contact, which must be as good as possible to allow for detecting the small voltages on the skin, occurring during a heartbeat cycle. Other related issues are connected with the well-known problems of conductive yarns and coatings, which often lose conductivity during washing and wearing. Here, recent solutions for these challenges are presented.

**Figure 1:**
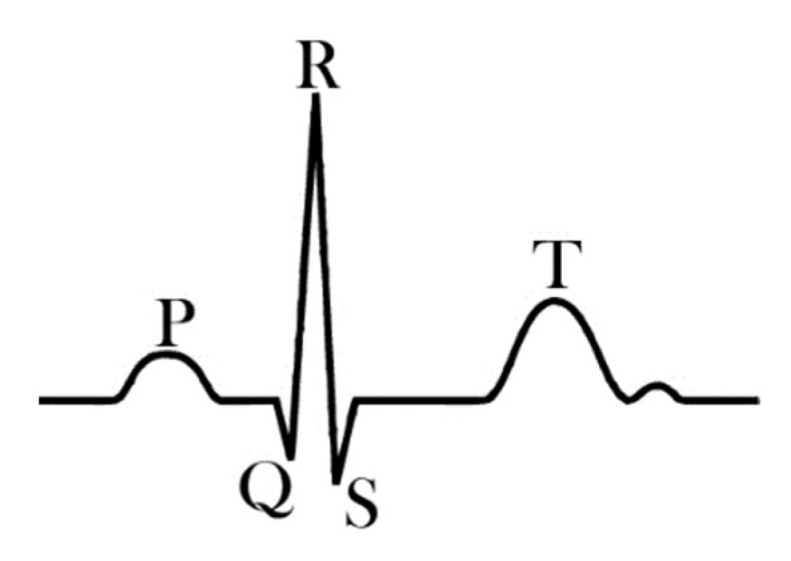
Typical features of an ECG signal. From Reference [18], originally published under a CC-BY license.

Generally, the ECG signal consists of a prominent QRS-complex, a preceding smaller P-wave and followed by a T-wave (Figure 1) [18]. While the detection of diverse illnesses of the heart necessitates investigating the full signal, including the heights of P- and T-waves, as well as the time distances between them and the QRS-complex, for other applications, it is sufficient to detect the pulse, i.e., the times at which the R-peak as the most prominent peak occurs. For this case, Alizadeh Meghrazi et al. suggested a multi-sensor system based on knitted conductive yarns which they applied on the waist, as opposed to the more common positioning at the chest. They developed a probabilistic approach to find R-peaks in noisy data, stemming from these textile electrodes, even if the probands were jogging, and suggested inserting textile electrodes in underwear, combined with this evaluation process, to measure the pulse constantly [18].

An interesting approach was chosen by Silva et al., who prepared ECG electrodes for the integration in swimsuits [19]. Here, the problems of surrounding water, as well as drag during swimming, had to be taken into account. They prepared textile electrodes from stainless-steel yarns by knitting different structures to reach an optimum skin contact and glued them on the proband’s skin with adhesive tape so that, at this stage, only the impact of water was taken into account. However, relaxed and stretched electrodes showed surprising functionality, since the signals measured in a state when the proband was submerged in water were even less noisy than measurements in dry state, which can be attributed to the improved skin contact due to the water film between skin and electrode. Muscular contractions and arm movement showed a strong impact on the detected signal, as it is usually found for measurements with textile electrodes and generally has to be filtered.

This problem of motion artifacts has been addressed in a recent study by An and Stylios [20]. They investigated the skin–electrode impedance for different durations and frequencies, using different knitted electrodes, and found a silver-plated knitted fabric of a defined minimum size to be ideal. In addition, they combined these textile electrodes with a miniaturized flexible printed circuit board with a motion sensor and found that both ECG and motion could be measured simultaneously in this way. However, no attempt was made to use the motion signals to reduce motion-related noise in the ECG signals.

While the previously reported textile ECG electrodes were knitted, elongation of these electrodes usually causes artifacts in the measured ECG signals. This is why Arquilla et al. used different sewn and embroidered electrodes [21]. The electrodes were prepared from silver-coated yarns, stitched onto an inextensible fabric and investigated by gluing them with tape on the chest of 8 probands. Interestingly, they found no significant differences between these textile electrodes and commercial gel-electrodes, regarding the detectability of the R-peak. Unfortunately, no unfiltered signals were shown, so that no evaluation of the P- and the T-wave was possible.

Besides the aforementioned silver-coated yarns and stainless-steel fiber or filament yarns, i.e., yarn made conductive by integration of metallic components, other materials can be used to make yarns or textile fabrics conductive. Carbon-based materials, such as carbon nanotubes (CNTs), carbon nanofibers or graphite coatings, are especially applied by several research groups. Lee and Cho combined single-walled CNTs with silver nanowires on a polyurethane nanoweb to produce ECG electrodes [22]. They found these electrodes to deliver suitable ECG signals on diverse probands, independent from their state of movement, body properties, etc., which they correlated with a low resistance of these conductive nanofabrics.

**Figure 2:**
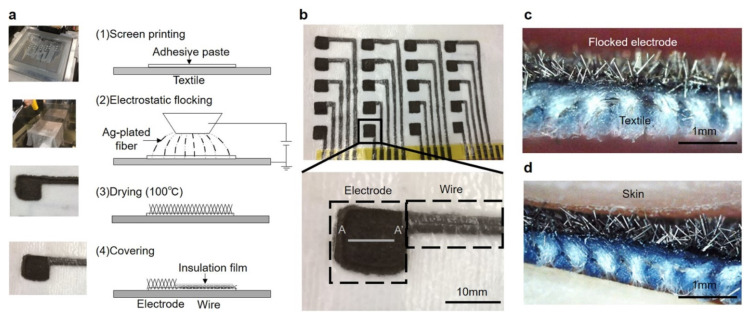
Production of flocked electrodes and wires with the electrostatic flocking technology. (**a**) Scheme of the production, with Ag-plated fivers being flocked on the textile with an adhesive paste; (**b**) photograph of electrodes and wires on a textile fabric; (**c**) cross-section view along the A-A′ line (Figure 2b) of a flocked electrode; and (**d**) flocked electrode in contact with the skin. From Reference [29], originally published under a CC-BY license.

Another point of differentiation is the number of leads applied in such textile-based systems. Typically, one or maximum two leads are used [23,24,25], with only few systems detecting 12-lead ECG signals [26,27,28], necessitating a minimum of 10 electrodes. Takeshita et al. used electrodes and wires with electrostatically flocked silver-plated fivers to produce arrays of conductive electrodes on textile fabrics (Figure 2) [29]. The conductive lines produced in this way were found to approximately double their resistance on an elongation of 40%, but the original values were restored after relaxation. During washing, the flocked wires showed a significantly smaller increase of the resistance than silver-paste wires. Using a skin phantom with an ECG generator, different pressures between 200 and 2000 Pa were found to result in good signal quality, depending on the displacement of the skin phantom with a frequency of 14/min, as to simulate electrode displacement due to breathing. These results were verified by using a real human proband. In this way, the authors could simultaneously measure 18 limb-ECG signals.

As mentioned before, ECG electrode can not only be produced by using conductive fibers or yarns, but also by conductive coatings, screen-printings, etc. Lidón-Roger et al., for example, used screen-printing to produce concentric ring electrodes consisting of an outer ring and an inner disk [30]. They used one conductive silver-based ink and a PEDOT:PSS (poly (3,4-ethylenedioxythiophene) polystyrene sulfonate), one of the most often used conductive polymers in the textile industry. ECG measurements applying only one of the ring electrodes, positioned on the chest, or both of them showed suitable signals for both conductive inks, with slightly better signal shapes for silver-based electrodes, for measuring on the left chest and between both positions.

Other authors reported on a variety of conductive coatings besides the aforementioned PEDOT:PSS [31,32], such as polypyrrole [33], graphene [34,35,36], graphite [37,38] or metals [39,40].

Finally, it should be mentioned that after the first attempts to measure ECG with textile electrodes, the progress found in the literature—or even translated into commercially available long-term ECG measurement systems with textile electrodes—is still limited. Several challenges have to be met, such as the generally bad electric contact from dry electrodes to the skin, undesired changes of the electric properties upon washing and wearing, even changes of the resistance due to stretching the electrodes, and last but not least displacements of the electrodes with respect to the body as soon as a proband moves or even breathes. Most recent attempts are aiming at solving one of these problems by improving either the textile structure or a conductive coating or by developing special clothing exerting sufficient pressure of the electrodes onto the skin to improve the electric contact. On the other hand, some groups try to develop evaluation algorithms which filter the important features from the usually noisy signals. 

However, as the large number of different attempts and the still limited commercial exploitation of the scientific results show, there is still a large gap to be closed between the recent state of textile ECG electrodes and the reliability necessitated in medical applications. Apparently, new ideas are necessary to solve these problems, either combining the knowledge of material scientists, textile engineers, electrical engineers, mathematicians, medical doctors and other disciplines, or finding completely new solutions, such as non-contact (i.e., typically capacitive) measurement methods [41,42,43], which recently pose other challenges to researchers. Anyway, textile ECG sensors are still a large and important research area, in spite of the steadily reported small progress.

## 3. Breathing Measurement

Breathing sensors are applied in sports or rehabilitation to monitor breathing patterns [44,45,46,47]. Breathing sensors have the advantage compared to ECG sensors that no direct skin contact is necessary; however, a fabric including a breathing sensor must still surround the chest in a closely fitting garment. This excludes fully woven or other non-extensible fabrics, so that often partly conductive knitted fabrics are used, or sensor threads or fibers are integrated into elastic fabrics. In the simplest version, breathing sensors can even be produced by integrating a rigid accelerometer into a chest belt [23], while most research groups aim at a higher level of textile integration [48].

Zięba and Frydrysiak, for example, investigated knitted and also woven fabrics containing conductive yarns, as well as optical fibers [49]. They found a nonlinear correlation of the electric resistance with the elongation for the fabrics containing conductive yarn, while the changes of the photodiode voltage, detected for the knitted system with optical fibers, showed a linear response, which is preferred in many applications. Nevertheless, the knitted fabric applied in a chest belt could also be used to clearly detect the breathing cycle of a proband. The optical sensor, consisting of a sender and a receiver waveguide, was developed further in a subsequent project and shown to work properly with diverse probands of different age [50], while the knitted resistance sensor was also modeled and experimentally investigated later on [51].

Carvalho et al. used knitted fabrics with different structures, including stainless-steel yarn and elastane, to prepare breathing sensors [52]. They found that stretching along the course direction resulted in reliable resistance changes with repeating waveform under cyclic stretching and relaxing, while elongation along the wale direction led to non-reproducible waveforms. Due to their experimental results, they suggested a pre-stretch of 30–50% to improve the repeatability of the measurements and a simple jersey structure to obtain reliable measurements.

Without elastane, different flat knitted structures from stainless-steel fiber yarn were investigated to optimize their usability as breathing sensors [53]. Strong differences were found depending on the knitted structure, the amount of conductive yarns and the stitch size, with full cardigan with small stitches showing only a small time-dependence of the resistance in an elongated state, while full cardigan with medium stitch size, stretched under a certain angle to the courses, all in all were optimum for breathing signal measurements.

Besides integration of conductive yarns or fibers into elastic textile fabrics, coating or screen-printing also belong to the technologies which can be applied to prepare textile breathing sensors. Furtak et al. presented a screen-printed breathing sensor, which has the advantage to be addable after shirt construction [54]. For this, they used a printing paste containing carbon nanotubes [55], applied on different shirts, and found a good correlation between sensor resistance and applied strain, as well as a relatively reliable possibility to measure breathing in this way, depending on the shirt [54].

A conductive silicone rubber containing carbon-black particles was used by Guo et al. to produce breathing sensors by knife-coating a double-faced elastic knitted fabric with the conductive material [56]. They found clear breathing patterns that were approximately identical with a commercial piezoelectric sensor used as a reference, and they managed extraction of breathing frequencies for a broad range of frequencies. Interestingly, differentiation between chest-dominated and abdominal-dominated breathing was also possible. Besides, in a simulation, it was possible to clearly detect sleep apnea. 

An interesting point in the production of not only prototypic, but really practically usable breathing sensors is their washability [57]. Berglund et al. investigated the effect of washing on textile stretching and bending sensors which were tested as pure textile sensors or insulated by a fusible polymer film [58]. Interestingly, they found not only a drift of the pure sensor upon washing and especially by tumble drying, but also delamination of the insulated films from the sensors, showing that not each insulation approach will work without problems.

While these studies were based on the elongation-dependent piezoresistive properties of textile fabrics, breathing measurements can also be performed by capacitive textile sensors [59,60], magnetic induction sensors [61] or fiber-optical sensors embedded in textile fabrics [62], as already mentioned for ECG measurements. While the latter can be integrated by typical textile techniques, as long as care is taken due to the high stiffness of even thin optical fibers, capacitive sensors can fully build up on a textile base, including not only the aforementioned conductive layers, but also the necessary insulators and dielectrics [63]. Such capacitive sensors can be produced by diverse textile techniques, not only by the aforementioned knitting processes, but also by sewing or embroidery [64].

Another challenge which occurs during the development of breathing sensors, similar to the aforementioned ECG sensors, is the differentiation between breathing signals and motion artifacts. Raiano et al. suggested an algorithm based on four piezoresistive textile sensors to allow for measuring rib cage movements, which were correlated with the simultaneous signals of an inertial measurement unit [65]. The authors showed that their algorithm could differentiate between breathing activity and torso rotation, as well as other movements due to running.

**Figure 3:**
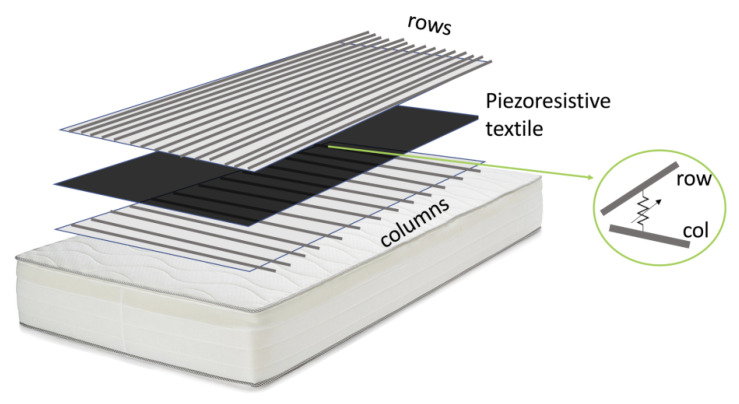
Three-layer piezoresistive array of 13 × 15 elements at the crossings between column and row conductors. From Reference [66], originally published under a CC-BY license.

Quite another idea to measure breathing especially during sleeping was most recently reported by Carbonaro et al. [66]. They integrated a matrix of nearly 200 piezoresistive sensors into a mattress to combine posture and movement classification with breathing detection (Figure 3). This matrix did not only enable detecting sleeping postures in the form of pressure images, but could also be used to estimate the breathing rate. For this, an algorithm was suggested which extracted this information from a local maximum of the power spectral density signal in the frequency domain.

Especially for the use in masks, as they are nowadays worn regularly, Li et al. suggested a graphdiyne-based printed flexible respiration sensor which works by detecting water molecules reaching the sensor during exhalation [67]. 

Finally, another point should not be forgotten here. Especially in case of knitted piezoresistive yarns, it is quite challenging, but also quite interesting in terms of basic research to model the electro-mechanical properties of stretched fabrics, in this way enabling a better orientation for researchers who strive for optimizing textile elongation sensors. Some research groups prepared corresponding electro-mechanical models, showing the influence of different knitted structures, resulting in different loop geometries and other network structures [68,69,70].

To conclude, several challenges formerly described for ECG electrodes—especially undesired modifications of conductive coatings or yarns due to washing or wearing—are also important in case of breathing sensors. Here, however, no skin contact is required, or more exactly, it is usually avoided. On the other hand, constant stretching or repeated stretching and relaxation can result in undesired wearing out, modifying the absolute resistance values with time. These issues have to be taken into account when textile breathing sensors are developed.

## 4. EMG Measurement

Textile electromyography (EMG) sensors are less often investigated than textile ECG sensors, but nevertheless can be found regularly in the literature. EMG sensors allow for measuring electrical muscle excitation, which is not only interesting in sports, but also for neuromuscular rehabilitation and control of prostheses [71,72,73]. Besides, completely different applications, such as computer controlling by facial electromyography, are reported in the literature [74].

Similar to textile ECG electrodes, there are possibilities to measure the EMG with or without skin contact. Linz et al., for example, investigated contactless EMG measurements, using a vest with embroidered circuits, connecting printed circuit boards (PCBs) with capacitive electrodes [75]. These contactless EMG sensors were connected with commercial glued EMG electrodes. The authors found good agreement of both sensors, with similar frequency-dependent voltage spectra. Finally, they showed full textile integration of the PCB into a textile circuit by embroidery.

Taelman et al. also suggested contactless EMG electrodes for stress analysis. They discussed the problems occurring due to displacement of the sensors embroidered in a shirt in relation to the desired position on a muscle that could result in erroneous measurements [76].

Pani et al., on the other hand, used textile electrodes in direct contact with the skin and compared them with commercial Ag/AgCl gel electrodes [77]. The textile electrodes were produced by screen-printing a PEDOT:PSS-based conductive paste onto a cotton fabric. Connections to the rigid electronics parts were performed by snap-buttons crimped on the fabric or by crocodile clamps, depending on the different measurements. They found a high repeatability of the screen-printing process, as evaluated by the measured sheet resistances of the textile sensors, but also a large skin–sensor impedance in case of completely dry textile electrodes. Nevertheless, the EMG signal quality was found suitable for measurements during walking, at least when the electrodes were repeatedly moisturized by saline solution.

Finni et al. decided to use conductive yarns instead of printed electrodes for contact EMG measurements [78]. They integrated electrodes consisting of conductive silver fiber and non-conductive synthetic yarns into knitted elastic shorts which positioned the electrodes in pairs on the distal quadriceps muscles, as well as reference electrodes longitudinally at the lateral sides. Similar to the aforementioned study by Pani et al., the electrodes were moisturized before the experiment. The results of these textile electrodes were found to be similar to those achieved by traditional gel electrodes; reproducibility was shown to be similar, or, in some cases, even higher, than for the commercial gel electrodes.

**Figure 4:**
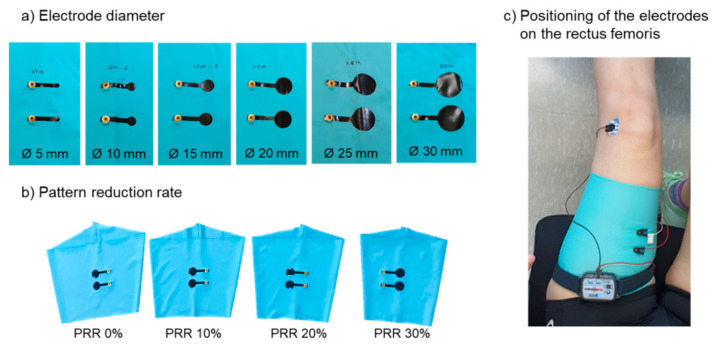
Electrode preparation in varied (**a**) electrode diameters and varied (**b**) pattern reduction rate (PRR). (**c**) positioning of the electroded on the rectus femoris. From Reference [79], originally published under a CC-BY license.

The influence of the electrode sizes was investigated by Kim et al., who prepared round bipolar EMG electrodes from carbon paste and silver paste on leg sleeves (Figure 4) [79]. Besides, they tested different clothing pressures, given by pattern reduction rates (PRRs) between 0 and 30%, as the leg sleeves showed only a width reduction without change of the length. They found a significant impact of the clothing pressure, suggesting a pressure of a minimum of 10 mmHg to reach a performance of the textile electrodes comparable to commercial Ag/AgCl electrodes. In addition, electrode diameters of a minimum of 20 mm were found to be optimum, since they showed reduced baseline noise and a better signal-to-noise ratio. The authors also mentioned that these results may differ for other textile electrodes prepared with different methods from other materials.

While the aforementioned studies mostly aimed at developing EMG sensors for sports, Lorussi et al. developed a complete textile platform for stroke patient treatment, including also EMG sensors [80]. The EMG electrodes were prepared from stainless-steel fiber yarns and included in square shape into the prototype garment to detect the activity of the deltoid muscle. To improve skin contact, a polyurethane foam and a neoprene back layer were applied behind the conductive layer. Connections were prepared by stainless-steel fiber cores with polyvinyl chloride (PVC) coating. The authors found these textile EMG electrodes to properly detect reaching activities of the probands.

Di Giminiani et al. combined EMG sensors with oximetry to monitor quadriceps activity during endurance and strength exercises [81]. For their experiments, they compared textile electrodes produced from silver-coated fabrics with a commercial gold-standard EMG and oximetry system and found especially the muscle crosstalk possibly captured by the textile electrodes to limit the application of textile electrodes. They mention the necessity to develop the integration design further in order to improve the skin/electrode contact.

As these examples show, EMG measurements by textile electrodes are generally possible, but they suffer from the same problems as ECG measurements, i.e., the high contact impedance between skin and electrode and the lack of shielding. While the first problem can be solved unambiguously in sports where the probands usually start sweating during exercises—in this way, significantly reducing the contact resistance—this solution is not suitable for applications in control of prostheses. Thus, more research is necessary in this field, too.

## 5. EEG Measurement

**Figure 5:**
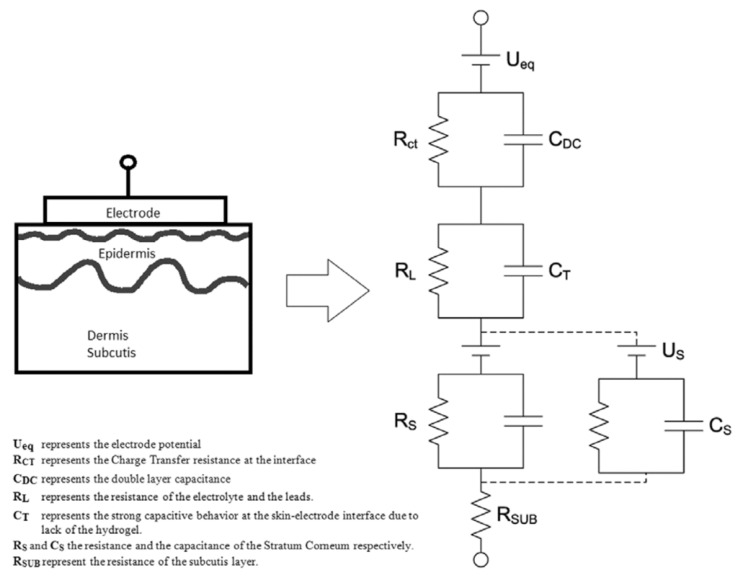
Equivalent circuit model for the skin–electrode interface. From Reference [83], originally published under a CC-BY license.

Besides ECG and EMG sensors, there are also attempts to use textile electrodes for electro-encephalography (EEG). Especially at the head, it is important to use soft electrodes to avoid pressure-points during sleeping [82]. Löfhede et al. prepared textile electrodes from a conductive knitted fabric with silver plating, pressed against the skin by a foam, and improved skin/electrode contact by adding a gel or saline solution [83]. They found similar signals for both kinds of electrodes, comparing slightly different yarns and knitted structures, and also no significant differences between gel and saline solution as a method to reduce the contact impedance. The contact impedance was modeled in their study by an equivalent circuit model dominated by capacitive behavior (Figure 5).

Reis Carneiro et al. used screen-printed EEG electrodes, including silver flakes, to improve the skin contact to a level comparable to standard gold Ag/AgCl or gold cup electrodes [84]. A second layer containing rigid PCB islands was used for contacting the electrodes, as well as to shield them from electromagnetic noise. In this way, the textile EEG sensors could be used for human machine interfaces (HMI) or sleep data acquisition.

Shu et al. designed the textile EEG electrodes to be sweat-absorbing in order to solve the problem of high skin/electrode impedance [85]. By using a multilayer textile fabric which includes not only a conductive layer, but also a sweat absorption layer, they could reduce the skin/electrode impedance and simultaneously reduce crosstalk interference between neighboring electrodes.

To prepare a therapeutic biofeedback system for young patients with autism spectrum disorder, Sahi et al. integrated textile-based EEG and EMG sensors into skull caps and baseball caps to enable unobtrusive monitoring of different brain patterns [86].

Several other groups investigated different textile electrodes to measure EEG signals for different purposes [87,88,89,90] or even developed a realistic, durable head phantom for the validation of EEG electrodes [91].

Nevertheless, it must be mentioned that Tseghai et al. reviewed recent approaches to produce textile-based EEG systems and pointed out severe problems with flexibility, stickability, washability and missing comparison with standard EEG systems, making the transfer of the scientific results into real commercial application hard [92]. These problems are equivalent to the aforementioned one, regarding other textile electrodes, although not explicitly mentioned in most reviews.

## 6. Bioimpedance Measurement

The last physical sensors based on conductive textile electrodes that are mentioned here are bioimpedance sensors. As the name already tells, bioimpedance measurements are performed with alternating current (AC) voltages and currents; that is, the aforementioned large capacitance (Figure 5) dominating the skin/electrode contact can be expected to modify the impedance values and the whole equivalent circuit model of such measurements. However, depending on textile structures, materials, temperatures, etc., different equivalent circuits will be representative to model the skin/electrode impedance [93]. Such bioimpedance measurements are often performed at a frequency of 50 kHz; alternatively, they can be applied as spectroscopic investigations.

Márquez et al. compared common Ag/AgCl electrodes with different textile electrodes [94]. They showed that the resistance spectra measured by the textile electrodes were reliable, while this was not the case for the reactance spectra taken with textile bracelet electrodes, but for reactance spectra measured with commercial textile cuffs. 

In a more recent project, the same group investigated the possibility of bioimpedance measurements along the chest by thorax and neck belts and compared the results of these textile electrode-based measurements with those of Ag/AgCl electrodes [95]. The aim of this study, however, was not to investigate the amount of water in the body, etc., but to test whether impedance cardiography could be used to detect a pulse signal. This was indeed shown to be possible, with ECG and bioimpedance measurements giving highly similar pulse rates for both kinds of electrodes. Besides, they investigated textile electrodes for body composition measurements and found them to be suitable for this purpose, too [96]. The investigation of body composition is of large importance to investigate possible overhydration of patients with chronic kidney disease or to monitor patients with lymphedema [97,98].

Going one step further, electrical impedance tomography (EIT) allows for imaging bone fracture repair, lung function, etc., in real time [99]. Jose et al. investigated EIT measurements with stretchable textile-based printed sensors and found the technique to be principally suitable for wearable imaging applications for health monitoring, although with relatively low resolution.

Besides these few examples of bioimpedance measurements using textile electrodes, diverse other groups report on various conductive textile electrodes used to investigate the human body composition or other impedance-related parameters of the human body [100,101,102,103,104]. However, here the large capacitance due to the high skin/electrode impedance and especially the possible variations of this value due to sweating or moving have to be taken into account even more than for ECG and similar measurements, especially since wrong bioimpedance signals due to ignoring the skin/body impedance are not recognized as easily as the typical noisy ECG signals found by measuring with not enough pressure on too dry skin.

## 7. Skin Temperature Measurement

Temperature can, in the easiest way, be measured by the resistance change in a conductive wire or by a thermocouple consisting of two wires from different metals. More sophisticated thermometers can also be integrated into textile fabrics since thermometers based on several physical effects can be implemented in miniaturized form, so that sometimes integration of a PT100, PTC (positive temperature coefficient) or NTC (negative temperature coefficient) sensor is easier and less error-prone than integration of a single wire whose conductivity may also be modified by washing and wearing, thus making such simple solutions not necessarily highly reliable.

**Figure 6:**
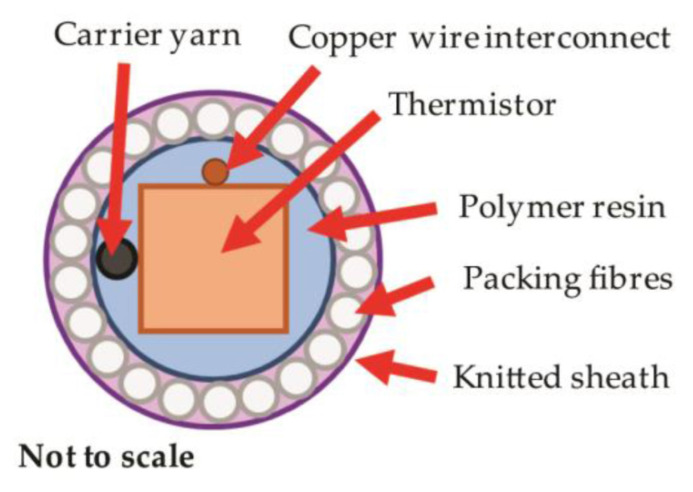
Cross-sectional scheme of thermistor encapsulated in a yarn comprising polymer resin, packing fibers and knitted sheath. The carrier yarn within the resin encapsulation improves the tensile strength of the final yarn. From Reference [105], originally published under a CC-BY license.

To prevent a temperature-sensing yarn from possible damage during washing, Hughes-Riley et al. encapsulated the thermistor (an NTC, i.e., a temperature-sensing semiconductor with negative temperature coefficient), as shown in Figure 6 [105]. These yarns were integrated into temperature-sensing socks, which would enable early warning of diabetic patients from impending ulcer formation and wound infection. They found very fast step response times when heating or cooling well below a second, while temperature changes only need to be measured on the order of hours. However, the accuracy of ±0.5 K should be improved by using more accurate temperature-sensing elements. Nevertheless, these yarns were found suitable to produce temperature measuring prototypes in the form of armbands, gloves or socks [106].

A similar approach of integrating a textile sensor in a yarn, this time by knit braiding, braiding and double covering, was chosen by Lugoda et al. [107]. Here the temperature-dependent layer was an Au layer deposited on a polyimide substrate. The authors found a slightly reduced sensitivity due to the reduced contact between temperature-dependent layer and the skin, with the best performance being shown by the thinnest yarn, i.e., the double-covering yarn. The thermal time constant for heating and cooling was below 10 s, which is, again, sufficient for measuring skin temperature due to medical reasons.

Another approach was chosen by Li et al., who integrated metal wires together with an elastic fabric circuit board into a woven fabric [108]. In this way, they prepared a highly deformable and elastic temperature sensor network with high sensitivity, accuracy and resolution, as well as short response time.

Wicaksoni et al. integrated diverse sensors into a sensing suit, including skin temperature detection, but also heart rate and respiration [109]. Here, the temperature sensor was also encapsulated by a thermally conductive epoxy and thermoplastic polyurethane. By comparing infrared thermography with the sensor readout, a calibration factor was defined by an offset and a linear multiplier to enable reliable temperature measurements.

Trung et al. developed elastomeric composite fibers which were capable of temperature measurement without being influenced by strain [110]. The stretchable fibers were composed of reduced graphene oxide (rGO)/polyurethane (PU) and implemented in a serpentine structure on a textile fabric. Response and thermal index of the device could be tailored by modifying the reduction time of the graphene oxide during the technological process. In this way, a stretchability of 90% could be reached with high temperature sensing resolution of 0.1 K and a high stability of ±0.37 K for an elongation from 0 to 50%.

As these examples show, many possibilities exist to produce textile temperature sensors. The requirements are partly lower than in the development of textile ECG, EMG, EEG, bioimpedance or similar sensors since the direct electrical skin contact is not necessary or, more exactly, not even desired. On the other hand, the influence of sweat, of washing and wearing is not negligible, and similar to the previously described bioimpedance sensors, deviations from the correct values are not necessarily recognized at once. Slow changes of the sensors, e.g., due to washing, may lead to undetected skin infections or other problems which should have been avoided by using textile temperature sensors. This is why there is still a large amount of new results reported in the recent scientific literature [111,112,113,114,115].

## 8. Moisture Detection

In the previous descriptions of all electrodes working with skin contact, the role of moisture on the quality of the detected signals due to the reduction of the skin/electrode impedance was mentioned. Moisture detection should thus be possible, in the simplest way, by measuring the impedance or, even easier, the direct current (DC) resistance between two conductive fibers or conduction lines in a defined distance. Indeed, this principle is adopted in many moisture sensors. Chen et al. combined it with a passive RFID (radio-frequency identification) tag to enable using the embroidered sensor without a battery [116,117].

Grethe et al. developed flexible, highly textile-integrated moisture sensors by spinning, printing and coating techniques [118]. They showed a sensitivity of these sensors in the range of 25–80% relative humidity and suggested their utilization in diverse areas, such as environmental control, medical textiles, working clothes or personal safety systems.

**Figure 7:**
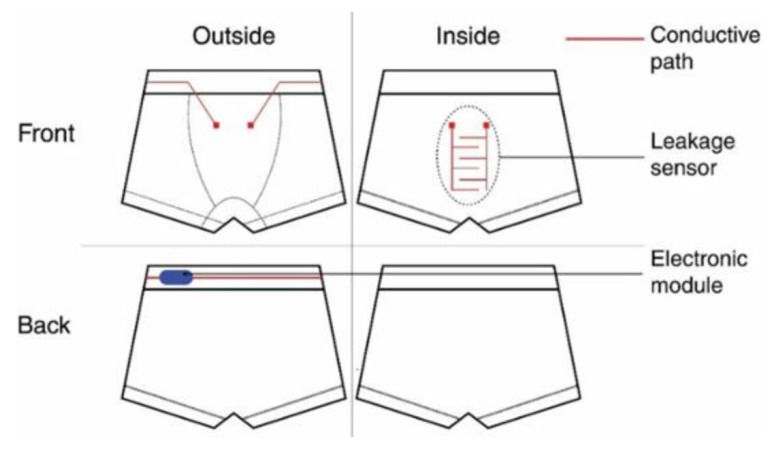
Scheme of the moisture-detecting underpants. From Reference [119], originally published under a CC-BY license.

For the special application of preventing children from nocturnal enuresis, Gaubert et al. developed underwear with textile moisture sensors which wake up the children by sound as soon as urine drops are detected [119]. The underpants were produced as two-layer system with a textile leakage sensor on the inner side linked to electronics on the outside, as shown in Figure 7, by conductive connections between inner and outer parts. The sensors were circular seamlessly knitted, including conductive silver-plated nylon yarns in a cotton/elastane substrate which was found ideal for moisture detection, while stainless-steel yarns with only 20% stainless steel could not properly detect leakage. Besides, the silver coating could withstand urine soiling and washing at 60 °C. To avoid degradation due to a current flow, the current was strictly limited, in this way increasing the system’s lifetime.

Similarly, Tanaka et al. constructed a diaper-shaped urine sensor with wireless transmitter [120]. Here, interestingly, the embedded battery consisted of absorbent material sandwiched between an aluminum and a carbon sponge electrode, allowing to receive 0.6 V which were up-converted to 2 V. In this way, they could transmit a signal over 5 m if a defined amount of urine is detected. The intermittent startup circuit used here to boost the input voltage and at the same time to suppress the quiescent current was developed further in a subsequent publication [121].

Pereira et al. investigated textile moisture sensors to prevent patients lying in bed or sitting in wheelchairs from pressure ulcers by integrating a conductive matrix in garments [122]. Using a multifilament yarn from poly(lactic acid) (PLA) with carbon nanotubes, Devaux et al. prepared a textile-based humidity sensor with good repeatability and fast reversibility [123]. Mecnika et al. prepared embroidered humidity sensors from conductive yarns [124], while Wendler et al. used multi-layer braiding to prepare a capacitive moisture sensor, working from 22 to 94% relative humidity at a high sensitivity [125]. 

As this short overview shows, textile-based moisture sensors are less problematic to produce than most of the aforementioned textile sensors. However, aging can occur in different ways, especially by oxidation of metallic parts, resulting in the necessity to recalibrate the sensors after washing or even after wearing to avoid erroneous results.

## 9. Sweat Examination

While the aforementioned sensors were mostly based on conductive materials and measurements of electrical parameters, sweat analysis is a more complicated task, usually combining chemical and physical properties. Here, only a brief overview of macroscopic textile-based sensors is given, while a recent discussion of electrospun electrochemical sensors can be found in [126].

Generally, diverse properties can be detected by an analysis of the human sweat, such as glucose, lactate, ascorbic acid [15], but also pH value and sweat rate during exercises [127]. Moreover, pH value measurements can be performed, for example, by conductive PEDOT:PSS coated fibers with an additional functionalization with bromothymol blue dye, as presented by Possanzini et al. [128]. The authors showed stable, reproducible and repeatable measurements for different yarn constructions, used in universal buffer and artificial sweat. Another way to create a textile-based pH sensor was suggested by Promphet et al. who used a color sensor with an indicator dye [129], while Caldara et al. used an organically modified silicate, including litmus and 3-glycidoxypropyltrimethoxysilane (GPTMS), as a siloxane precursor to detect pH levels also via a color sensor [130]. 

Besides colorimetric sensors which are often applied in sweat analysis [131,132,133,134,135], there are also other types of sensors based on measuring the electrical properties of specifically functionalized conductive fibers, yarns or textile fabrics, which are changed upon contact of the functionalized sensor with a defined molecule, etc. [136,137,138,139,140].

## 10. Advantages and Challenges of Textile-Based Biosensors

As this review shows, there are diverse textile-based sensors under investigation to make biosignal detection easier. However, it is necessary to mention not only the advantages, but also the challenges which still have to be met and may, due to physical limitations, never be overcome.

On the one hand, especially regarding sensors touching the skin, textile sensors have large advantages for long-term measurements. They can be made in a highly skin-friendly, elastic way so that such ECG and other sensors can be worn for long durations without impeding the proband. In other cases, textile sensors which have to be worn next to the body, but not necessarily in direct contact to the skin—such as breathing sensors—still have the advantage of causing less reduction of the wearing comfort than a rigid, stiff sensor. This is why there are already some sensor shirts on the market measuring biosignals [141,142].

Another advantage of textile sensors is their reusability—opposite to disposable glued sensors, e.g., for ECG or other measurements with direct skin contact, they can, in principle, be washed and used again, making them not only more sustainable, but sometimes even less expensive.

On the other hand, there are several drawbacks of textile-based biosensors. In case of sensors touching the skin, such as ECG, the skin contact is crucial. The skin–sensor impedance is reduced by sweating so that these sensors can be used well for athletes, firefighters on duty, etc.; however, especially long-term monitoring of elderly people who often have dry skin is much more challenging. With increased skin–sensor impedance, the common filtering step used in long-term ECG measurements where probands move—especially important during sports and other strong movements—becomes more complicated and thus less reliable so that single events, such as single missing or doubled heartbeats, may stay undetected. This is uncritical as long as the average pulse is to be measured, but may become critical for sportsmen, firefighters or other people who have to be examined closely during highly exhausting work.

Another problem of fully textile solutions is the often missing electromagnetic shielding of electrodes and connections, the latter often being realized by conductive yarns and normally not shielded. This can not only be problematic if people are working near machines with electromagnetic radiation, but also for hospitalized people who are also in an environment where electromagnetic noise occurs due to the surrounding measurement equipment.

To conclude, the textile sensors described in this review have several advantages if they are used for long-term monitoring. For short-term measurements of ECG, etc., in a doctor’s office, they would be too unreliable and too expensive, especially since their high skin–sensor impedance impedes using them with common electronic measurement equipment used for gel electrodes. Some of the aforementioned problems of the sensors which are not in direct skin contact will surely be solved by further research, while the high skin–sensor impedance of ECG and similar sensors necessitates not only further research on hydrogel-impregnated textile electrodes, etc., but also improved electronic filters to enable monitoring even single events in noisy signals.

## 11. Conclusions

To conclude, diverse physical and partly chemical properties of humans can be measured by textile sensors. Most of them are based on measuring electric properties, such as resistance, impedance, voltage or capacity, which are enabled by conductive fibers, yarns or coatings, and sometimes also by fine metal wires. The main challenges in developing textile biosensors are as follows: the high contact impedance between dry skin and textile electrode; if the sensors detect their signals directly on the skin; and undesired changes of the resistance and other electric properties of the sensors, due to washing and wearing. 

While a large number of research groups work on developing further such textile biosensors, these main challenges are still unsolved, often impeding the transfer from lab into industry. As this brief overview shows, there are still many challenges to solve, either by innovative combinations of interdisciplinary solutions or by completely new approaches to measure these long-investigated biosignals with different sensor principles. 

## Data Availability

In this review paper, no new data were created.

## References

[1] Pantelopoulos A., Bourbakis N. A survey on wearable biosensor systems for health monitoring. Proceedings of the 2008 30th Annual Conference of the IEEE Engineering in Medicine and Biology Society.

[2] Schwarz A., van Langenhove L., Guermonprez P., Deguillemont D. (2010). A roadmap on smart textiles. Text. Progress.

[3] Stoppa M., Chiolerio A. (2014). Wearable electronics and smart textiles: A critical review. Sensors.

[4] Omura Y., Hashmi S. (2014). Sensor Technology for Monitoring of Health-Related Conditions. Comprehensive Materials Processing; Sensor Materials, Technologies and Applications.

[5] Koncar V. (2016). Introduction to smart textile and their applications. Smart Textiles and their Applications.

[6] Ehrmann G., Ehrmann A. (2021). Electronic textiles. Encyclopedia.

[7] Hughes-Riley T., Dias T., Cork C. (2018). A historical review of the development of electronic textiles. Fibers.

[8] Wagner S., Bonderover E., Jordan W.B., Sturm J.C. (2002). Electrotextiles: Concepts and challenges. Int. J. High Speed Electron. Syst..

[9] Ehrmann G., Ehrmann A. (2020). Suitability of common single circuit boards for sensing and actuating in smart textiles. Commun. Dev. Assem. Text. Prod..

[10] Paiva A., Ferreira F., Catarino A., Carvalho M., Carvalho H. (2018). Design of smart garments for sports and rehabilitation. IOP Conf. Ser. Mater. Sci. Eng..

[11] Khundaqji H., Hing W., Furness J., Climstein M. (2020). Smart shirts for monitoring physiological parameters: Scoping review. JMIR Mhealth Uhealth.

[12] Scataglini S., Moorhead A.P., Feletti F. (2020). A systematic review of smart clothing in sports: Possible applications to extreme sports. Muscles Ligaments Tendons J..

[13] Bartlett P.N., Archer P.B.M., Ling-Chung S.K. (1989). Conducting polymer gas sensors part I: Fabrication and characterization. Sens. Actuators.

[14] Han J.-W., Kim B.S., Li J., Meyyappan M. (2013). A carbon nanotube based ammonia sensor on cotton textile. Appl. Phys. Lett..

[15] He W., Wang C., Wang H.M., Jian M.Q., Lu W.D., Liang X.P., Zhang X., Yang F.C., Zhang Y.Y. (2019). Integrated textile sensor patch for real-time and multiplex sweat analysis. Sci. Adv..

[16] Angelucci A., Cavicchioli M., Cintorrino I.A., Lauricella G., Rossi C., Strati S., Aliverti A. (2021). Smart textiles and sensorized garments for physiological monitoring: A review of available solutions and techniques. Sensors.

[17] Kamarudin S.F., Mustapha M., Kim J.-K. (2021). Green strategies to printed sensors for healthcare applications. Polymer Rev..

[18] Meghrazi M.A., Tian Y., Mahnam A., Bhattachan P., Eskandarian L., Kakhki S.T., Popovic M.R., Lankarany M. (2020). Multichannel ECG recording from waist using textile sensors. BioMed. Eng. OnLine.

[19] Silva M., Catarino A., Carvalho H., Rocha A., Monteiro J., Montagna G. Textile sensors for ECG and respiratory frequency on swimsuits. Proceedings of the Intelligent Textiles and Mass Customisation International Conference.

[20] An X., Stylios G.K. (2018). A hybrid textile electrode for electrocardiogram (ECG) measurement and motion tracking. Materials.

[21] Arquilla K., Webb A.K., Anderson A.P. (2020). Textile electrocardiogram (ECG) electrodes for wearable health monitoring. Sensors.

[22] Lee E., Cho G.S. (2019). PU nanoweb-based textile electrode treated with single-walled carbon nanotube/silver nanowire and its application to ECG monitoring. Smart Mater. Struct..

[23] Jourand P., Clercq H.D., Corthout R., Puers R. (2009). Textile Integrated Breathing and ECG Monitoring System. Procedia Chem..

[24] Cho G., Jeong K., Paik M.J., Kwun Y., Sung M. (2011). Performance evaluation of textile-based electrodes and motion sensors for smart clothing. IEEE Sens. J..

[25] Rai P., Kumar P.S., Oh S.C., Kwon H.J., Mathur G.N., Varadan V.K., Agarwal M.P. (2012). Smart healthcare textile sensor system for unhindered-pervasive health monitoring. Proc. SPIE.

[26] Bouwstra S., Chen W., Oetomo S.B., Feijs L.M.G., Gluitmans P.J.M. Designing for reliable textile neonatal ECG monitoring using multi-sensor recordings. Proceedings of the 2011 Annual International Conference of the IEEE Engineering in Medicine and Biology Society.

[27] Wiklund U., Karlsson M., Östlund N., Berlin L., Lindecrantz K., Karlsson S., Sandsjö L. (2007). Adaptive spatio-temporal filtering of disturbed ECGs: A multi-channel approach to heartbeat detection in smart clothing. Med. Biol. Eng. Comput..

[28] Trummer S., Ehrmann A., Büsgen A. (2017). Development of underwear with integrated 12 channel ECG for men and women. AUTEX Res. J..

[29] Takeshita T., Yoshida M., Takei Y., Ouchi A., Hinoki A., Uchida H., Kobayashi T. (2019). Relationship between contact pressure and motion artifacts in ECG measurements with electrostatic flocked electrodes fabricated on textile. Sci. Rep..

[30] Lidón-Roger J.V., Prats-Boluda G., Ye-Lin Y.Y., Garcia-Casado J., Garcia-Breijo E. (2018). Textile concentric ring electrodes for ECG recording based on screen-printing technique. Sensors.

[31] Ankhili A., Tao X.Y., Cochrane C., Koncar V., Coulon D., Tarlet J.-M. (2019). Ambulatory evaluation of ECG signals obtained using washable textile-based electrodes made with chemically modified PEDOT:PSS. Sensors.

[32] Ankhili A., Tao X.Y., Cochrane C., Koncar V., Coulon D., Tarlet J.-M. (2018). Comparative study on conductive knitted fabric electrodes for long-term electrocardiography monitoring: Silver-plated and PEDOT:PSS coated fabrics. Sensors.

[33] Wattal S.S., Spear S.K., Imtiaz M.H., Sazonov E. A polypyrrole-coated textile electrode and connector for wearable ECG monitoring. Proceedings of the 2018 IEEE International Conference on Wearable and Implantable Body Sensor Networks (BSN).

[34] Akter Shathi M., Chen M.Z., Khoso N.A., Rahman M.T., Bhattacharjee B. (2020). Graphene coated textile based highly flexible and washable sports bra for human health monitoring. Mater. Des..

[35] Yapici M.K., Aklhidir T., Samad Y.A., Liao K. (2015). Graphene-clad textile electrodes for electrocardiogram monitoring. Sens. Actuators A Chem..

[36] Xu X.W., Luo M., He P., Guo X.J., Yang J.L. (2019). Screen printed graphene electrodes on textile for wearable electrocardiogram monitoring. Appl. Phys. A.

[37] Aumann S., Trummer S., Brücken A., Ehrmann A., Büsgen A. (2014). Conceptual design of a sensory shirt for fire-fighters. Text. Res. J..

[38] Schäl P., Junger I.J., Grimmelsmann N., Ehrmann A. (2018). Development of graphite-based conductive textile coatings. J. Coat. Technol. Res..

[39] Weder M., Hegemann D., Amberg M., Hess M., Boesel L.F., Abächerli R., Meyer V.R., Rossi R.M. (2015). Embroidered electrode with silver/titanium coating for long-term ECG monitoring. Sensors.

[40] Nigusse A.B., Malengier B., Mengistie D.A., van Langenhove L. Evaluation of silver-coated textile electrodes for ECG recording. Proceedings of the 2021 IEEE International Conference on Flexible and Printable Sensors and Systems (FLEPS).

[41] Fong E.-M., Chung W.-Y. (2015). A hygroscopic sensor electrode for fast stabilized non-contact ECG signal acquisition. Sensors.

[42] Majumder S., Chen L., Marinov O., Chen C.-H., Mondal T., Deen M.J. (2018). Noncontact wearable wireless ECG systems for long-term monitoring. IEEE Rev. Biomed. Eng..

[43] Wang T.-W., Zhang H., Lin S.-F. (2020). Influence of capacitive coupling on high-fidelity non-contact ECG measurement. IEEE Sens. J..

[44] Mitchell E., Coyle S., O’Connor N., Diamond D., Ward T. Breathing feedback system with wearable textile sensors. Proceedings of the 2010 IEEE International Conference on Body Sensor Networks.

[45] Guo L., Berglin L., Li Y.J., Mattila H., Kalantar Mehrjerdi A., Skrifvars M. ‘Disappearing sensor’-textile based sensor for monitoring breathing. Proceedings of the 2011 International Conference on Control, Automation and Systems Engineering (CASE).

[46] Ramos-Garcia R.I., da Silva F., Kondi Y., Sazonov E., Dunne L.E. Analysis of a coverstitched stretch sensor for monitoring of breathing. Proceedings of the 2016 10th International Conference on Sensing Technology (ICST).

[47] Rovira C., Coyle S., Corcoran B., Diamond D., Stroiescu F., Daly K. Integration of textile-based sensors and Shimmer for breathing rate and volume measurement. Proceedings of the 2011 5th International Conference on Pervasive Computing Technologies for Healthcare (PervasiveHealth) and Workshops.

[48] Yang C.-M., Huang W.-T., Yang T.-L., Hsieh M.-C., Liu C.-T. Textile digital sensors for detecting breathing frequency. Proceedings of the 2008 5th International Summer School and Symposium on Medical Devices and Biosensors.

[49] Zięba J., Frydrysiak M. (2006). Textronics–electrical and electronic textiles. Sensors for breathing frequency measurement. Fibres Text. East. Eur..

[50] Zięba J., Frydrysiak M., Gniotek K. (2007). Textronics system for breathing measurement. Fibres Text. East. Eur..

[51] Zięba J., Frydrysiak M., Błaszczyk J. Textronic clothing with resistance textile sensor to monitoring frequency of human breathing. Proceedings of the 2012 IEEE International Symposium on Medical Measurements and Applications.

[52] Carvalho H., Catarino A.P., Rocha A., Postolache O. Health monitoring using textile sensor and electrodes: An overview and integration of technologies. Proceedings of the 2014 IEEE International Symposium on Medical Measurements and Applications (MeMeA).

[53] Ehrmann A., Heimlich F., Brücken A., Weber M.O., Haug R. (2014). Suitability of knitted fabrics as elongation sensors subject to structure, stitch dimension and elongation direction. Text. Res. J..

[54] Furtak N.T., Skrzetuska E., Krucinska I. (2013). Development of screen-printed breathing rate sensor. Fibres Text. East. Eur..

[55] Krucińska I., Skrzetuska E., Urbaniak-Domagala W. (2012). Prototypes of carbon nanotube-based textile sensors manufactured by the screen printing method. Fibres Text. East. Eur..

[56] Guo L., Berglin L., Wiklund U., Mattila H. (2012). Design of a garment-based sensing system for breathing monitoring. Text. Res. J..

[57] Zeagler C., Gilliland S., Audy S., Starner T. Can I wash it? The effect of washing conductive materials used in making textile based wearable electronic interfaces. Proceedings of the 2013 International Symposium on Wearable Computers.

[58] Berglund M.E., Coughlin J., Gioberto G., Dunne L.E. (2014). Washability of E-Textile Stretch Sensors and Sensor Insulation. Proceedings of the 2014 ACM International Symposium on Wearable Computers.

[59] Kang T.-H., Merritt C., Karaguzel B., Wilson J., Franzon P., Pourdeyhimi B., Grant E., Nagle T. Sensors on textile substrates for home-based healthcare monitoring. Proceedings of the 1st Transdisciplinary conference on Distributed Diagnosis and Home Healthcare.

[60] Min S.D., Yun Y.Y., Shin H.S. (2014). Simplified structural textile respiration sensor based on capacitive pressure sensing method. IEEE Sens. J..

[61] Teichmann D., Foussier J., Buscher M., Walter M., Leonhardt S. Textile integration of a magnetic induction sensor for monitoring of cardiorespiratory activity. Proceedings of the World Congress on Medical Physics and Biomedical Engineering.

[62] Yang X.F., Chen Z.H., Ming Elvin C.S., Ying Janice L.H., Ng S.H., Teo J.T., Wu R.F. (2015). Textile fiber optic microbend sensor used for heartbeat and respiration monitoring. IEEE Sens. J..

[63] Yang C.M., Yang T.L., Wu C.C., Hung S.H., Liao M.H., Su M.J., Hsieh H.C. Textile-based capacitive sensor for a wireless wearable breath monitoring system. Proceedings of the 2014 IEEE International Conference on Consumer Electronics (ICCE).

[64] Agcayazi T., Yokus M.A., Gordon M., Ghosh T., Bozkurt A. A stitched textile-based capacitive respiration sensor. Proceedings of the 2017 IEEE Sensors.

[65] Raiano L., di Tocco J., Massaroni C., di Pino G., Schena E., Formica D. Clean-breathing: A novel sensor fusion algorithm based on ICA to remove motion artifacts from breathing signal. Proceedings of the 2020 IEEE International Workshop on Metrology for Industry 4.0 & IoT.

[66] Carbonaro N., Laurino M., Arcarisi L., Menicuddi D., Gemignani A., Tognetti A. (2021). Textile-based pressure sensing matrix for in-bed monitoring of subject sleeping posture and breathing activity. Appl. Sci..

[67] Li Y., Zhang M.J., Hu X.L., Yu L.M., Fan X.H., Huang C.S., Li Y.L. (2021). Graphdiyne-based flexible respiration sensors for monitoring human health. NanoToday.

[68] Atalay O., Kennon W.R., Demirok E. (2015). Weft-knitted strain sensor for monitoring respiratory rate and its electro-mechanical modeling. IEEE Sens. J..

[69] Bahadir S.K. (2018). Identification and modeling of sensing capability of force sensing resistor integrated to e-textile structure. IEEE Sens. J..

[70] Ejupi A., Ferrone A., Menon C. Quantification of textile-based stretch sensors using machine learning: An exploratory study. Proceedings of the 2018 7th IEEE International conference on Biomedical Robotics and Biomechatronics (Biorob).

[71] Zhang H.S., Zhang L.Q., Li G.L. Using textile electrode EMG for prosthetic movement identification in transradial amputees. Proceedings of the 2013 IEEE International Conference on Body Sensor Networks.

[72] Niijima A., Isezaki T., Aoki R., Watanabe T., Yamada T. hitoeCap: Wearable EMG sensor for monitoring masticatory muscles with PEDOT-PSS textile electrodes. Proceedings of the 2017 ACM International Symposium on Wearable Computers.

[73] Di Giminiani R., Lancia S., Ferrari M., Quaresima V., Tilma Vistisen H., Kliltgaard A., Arbjerg Heick R., Oestergard K., Yndgaard Soerensen K., Cardinale M. A wearable integrated textile EMG and muscle oximetry system for monitoring exercise-induced effects: A feasibility study. Proceedings of the 2018 IEEE International Symposium on Medical Measurements and Applications (MeMeA).

[74] Paul G.M., Cao F., Torah R., Yang K., Beeby S., Tudor J. (2014). A smart textile based facial EMG and EOG computer interface. IEEE Sens. J..

[75] Linz T., Gourmelon L., Langereis G., Leonhardt S., Falck T., Mähönen P. (2007). Contactless EMG sensors embroidered onto textile. 4th International Workshop on Wearable and Implantable Body Sensor Networks (BSN 2007).

[76] Taelman J., Adriaensen T., van Der Horst C., Linz T., Spaepen A. Textile integrated contactless EMG sensing for stress analysis. Proceedings of the 2007 29th Annual International conference of the IEEE Engineering in Medicine and Biology Society.

[77] Pani D., Achilli A., Spanu A., Bonfiglio A., Gazzoni M., Botter A. (2019). Validation of polymer-based screen-printed textile electrodes for surface EMG detection. IEEE Trans. Neural Syst. Rehabil. Eng..

[78] Finni T., Hu M., Kettunen P., Vilavuo T., Cheng S. (2007). Measurement of EMG activity with textile electrodes embedded into clothing. Physiol. Meas..

[79] Kim S.Y., Lee S.J., Jeong W.Y. (2020). EMG measurement with textile-based electrodes in different electrode sizes and clothing pressures for smart clothing design optimization. Polymers.

[80] Lorussi F., Carbonaro N., de Rossi D., Paradiso R., Veltink P., Tognetti A. (2016). Wearable textile platform for assessing stroke patient treatment in daily life conditions. Front. Bioeng. Biotechnol..

[81] Di Giminiani R., Cardinale M., Ferrari M., Quaresima V. (2020). Validation of fabric-based thigh-wearable EMG sensors and oximetry for monitoring quadriceps activity during strength and endurance exercises. Sensors.

[82] Löfhede J., Seoane F., Thordstein M. Soft textile electrodes for EEG monitoring. Proceedings of the 10th IEEE International Conference on Information Technology and Applications in Biomedicine.

[83] Löfhede J., Seoane F., Thordstein M. (2012). Textile electrodes for EEG recording–a pilot study. Sensors.

[84] Reis Carneiro M., de Almeida A.T., Tavakoli M. (2020). Wearable and comfortable e-textile headband for long-term acquisition of forehead EEG signals. IEEE Sens. J..

[85] Shu L., Xu T.Y., Xu X.M. (2019). Mulilayer sweat-absorbable textile electrode for EEG measurement in forehead site. IEEE Sens. J..

[86] Sahi A., Rai P., Oh S.C., Ramasamy M., Harbauth R.E., Varadan V.K. (2014). Neural activity based biofeedback therapy for Autism spectrum disorder through wearable wireless textile EEG monitoring system. Proc. SPIE.

[87] (2013). Nano-bio-textile sensors with mobile wireless platform for wearable health monitoring of neurological and cardiovascular disorders. J. Electrochem. Soc..

[88] Asl S.N., Ludwig F., Schilling M. (2015). Nose properties of textile, capacitive EEG electrodes. Curr. Dir. Biomed. Eng..

[89] Fleury A., Alizadeh M., Stefan G., Chau T. Toward fabric-based EEG access technologies: Seamless knit electrodes for a portable brain-computer interface. Proceedings of the 2017 IEEE Life Sciences Conference.

[90] Tseghai G.B., Malengier B., Fante K.A., van Langenhove L. A dry EEG textrode from a PEDOT:PSS/PDMS-coated cotton fabric for brain activity monitoring. Proceedings of the 2021 IEEE International Conference on Flexible and Printable Sensors and Systems (FLEPS).

[91] Tseghai G.B., Malengier B., Fante K.A., van Langenhove L. (2021). A long-lasting textile-based anatomically realistic head phantom for validation of EEG electrodes. Sensors.

[92] Tseghai G.B., Malengier B., Fante K.A., van Langenhove L. (2021). The status of textile-based dry EEG electrodes. AUTEX Res. J..

[93] Medrano G., Ubl A., Zimmermann N., Gries T., Leonhardt S., Scharfetter H., Merwa R. (2007). Skin electrode impedance of textile electrodes for bioimpedance spectroscopy. 13th International Conference on Electrical Bioimpedance and the 8th Conference on Electrical Impedance Tomography.

[94] Márquez J.C., Seoane F., Välimäki E., Lindecrantz K. (2010). Comparison of dry-textile electrodes for electrical bioimpedance spectroscopy measurements. J. Phys. Conf. Ser..

[95] Márquez J.C., Seoane F., Välimäki E., Lindecrantz K. (2013). Textrode-enabled transthoracic electrical bioimpedance measurements –towards wearable applications of impedance cardiography. J. Electr. Bioimpedance.

[96] Márquez J.C., Seoane F., Lindecrantz K. (2013). Textrode functional straps for bioimpedance measurements–experimental results for body composition analysis. Eur. J. Clin. Nutr..

[97] Ferreira J., Pau I., Lindecrantz K., Seoane F. (2017). A handheld and textile-enabled bioimpedance system for ubiquitous body composition analysis. An initial functional validation. IEEE J. Biomed. Health Inform..

[98] Meding J.T., Tuvshinbayar K., Döpke C., Tamoue F. (2021). Textile electrodes for bioimpedance measuring. Commun. Dev. Assem. Text. Prod..

[99] Jose M., Lemmens M., Bormans S., Thoelen R., Deferme W. (2021). Fully printed, stretchable und wearable bioimpedance sensor on textiles for tomography. Flex. Print. Electron..

[100] Medrano G., Beckmann L., Zimmermann N., Grundmann T., Gries T., Leonhardt S., Leonhardt S., Falck T., Mähönen P. (2007). Bioimpedance spectroscopy with textile electrodes for a continous monitoring application. 4th International Workshop on Wearable and Implantable Body Sensor Networks (BSN 2007).

[101] Posada-Quintero H.F., Reljin N., Eaton-Robb C., Noh Y.S., Riistama J., Chon K.H. (2018). Analysis of consistency of transthoracic bioimpedance measurements acquired with dry carbon black PDMS electrodes, adhesive electrodes, and wet textile electrodes. Sensors.

[102] Corchia L., Monti G., Raheli F., Candelieri G., Tarricone L. (2020). Dry textile electrodes for wearable bio-impedance analyzers. IEEE Sens. J..

[103] Ulbrich M., Lüken M., Mühlsteff J., Leonhardt S. (2021). Wearable bioimpedance systems for home-care monitoring using BSNs. Wearable Sensors.

[104] Pavlin M., Novak F., Papa G. (2021). Low power contactless bioimpedance sensor for monitoring breathing activity. Sensors.

[105] Hughes-Riley T., Lugoda P., Dias T., Trabi C.L., Morris R.H. (2017). A study of thermistor performance within a textile structure. Sensors.

[106] Lugoda P., Hughes-Riley T., Oliveira C., Morris R., Dias T. (2018). Developing novel temperature sensing garments for health monitoring applications. Fibers.

[107] Lugoda P., Costa J.C., Oliveira C., Garcia-Garcia L.A., Wickramasinghe S.D., Pouryazdan A., Roggen D., Dias T., Münzenrieder N. (2020). Flexible temperature sensor integration into e-textiles using different industrial yarn fabrication processes. Sensors.

[108] Li Q., Chen H., Ran Z.-Y., Zhang L.-N., Xiang R.-F., Wang X., Tao X.-M., Ding X. (2018). Full fabric sensing network with large deformation for continuous detection of skin temperature. Smart Mater. Struct..

[109] Wicaksoni I., Tucker C.I., Sun T., Guerrero C.A., Liu C., Wo W.M., Pence E.J., Dagdeviren C. (2020). A tailored, electronic textile conformable suit for large-scale spatiotemporal physiological sensing in vivo. Npj Flex. Electron..

[110] Trung T.Q., Dang T.M.L., Ramasundaram S., Toi P.T., Park S.Y., Lee N.-E. (2019). A stretchable strain-insensitive temperature sensor based on free-standing elastomeric composite fibers for on-body monitoring of skin temperature. ACS Appl. Mater. Interfaces.

[111] Lugoda P., Hughes-Riley T., Morris R., Dias T. (2018). A wearable textile thermograph. Sensors.

[112] Trung T.Q., Le H.S., Dang T.M.L., Ju S.H., Park S.Y., Lee N.-E. (2018). Freestanding, fiber-based, wearable temperature sensor with tunable thermal index for healthcare monitoring. Adv. Healthc. Mater..

[113] Wu R.H., Ma L.Y., Hou C., Meng Z.H., Guo W.X., Yu W.D., Yu R., Hu F., Liu X.Y. (2019). Silk composite electronic textile sensor for high space precision 2D combo temperature-pressure sensing. Small.

[114] Hughes-Riley T., Jobling P., Dias T., Faulkner S.H. (2021). An investigation of temperature-sensing textiles for temperature monitoring during sub-maximal cycling trials. Text. Res. J..

[115] Wang Y.B., Zhu M.M., Wie X.D., Yu J.Y., Li Z.L., Ding B. (2021). A dual-mode electronic skin textile for pressure and temperature sensing. Chem. Eng. J..

[116] Chen X.C., He H., Khan Z., Sydanheimo L., Ukkonen L., Virkki J. (2020). Textile-based batteryless moisture sensor. IEEE Antennas Wirel. Propag. Lett..

[117] Chen X.C., He H., Khan Z., Sydänheimo L., Ukkonen L., Virkki J. Design, fabrication and wireless evaluation of a passive 3D-printed moisture sensor on a textile substrate. Proceedings of the 2019 Photonics & Electromagnetics Research Symposium–Spring (PIERS-Spring).

[118] Grethe T., Borczyk S., Plenkmann K., Normann M., Rabe M., Schwarz-Pfeiffer A. Textile humidity sensor. Proceedings of the 2018 Symposium on Design, Test, Integration & Packaging of MEMS and MOEMS (DTIP).

[119] Gaubert V., Gidik H., Koncar V. (2020). Boxer underwear incorporating textile moisture sensor to prevent nocturnal enuresis. Sensors.

[120] Tanaka M., Utsunomiya F., Douseki T. (2016). Wearable self-powered diaper-shaped urinary-incontinence sensor suppressing response-time variation with 0.3 V start-up converter. IEEE Sens. J..

[121] Sudo M., Utsunomiya F., Tanaka M., Douseki T. (2021). Temperature-robuts 0.48-V FD-SOI intermittent startup circuit with 300-nA quiescent current for batteryless wireless sensor capable of 1-µA energy harvesting sources. IEICE Trans..

[122] Pereira T., Silva P., Carvalho H., Carvalho M. Textile moisture sensor matrix for monitoring of disabled and bed-rest patients. Proceedings of the 2011 IEEE Eurocon–International Conference on Computer as a Tool.

[123] Devaux E., Aubry C., Campagne C., Rochery M. (2011). PLA/carbon nanotubes multifilament yarns for relative humidity textile sensor. J. Eng. Fibers Fabr..

[124] Mecnika V., Hoerr M., Jockenhoefel S., Gries T., Krievins I., Schwarz-Pfeiffer A. Preliminary study on textile humidity sensors. Proceedings of the Smart SysTech 2015–European Conference on Smart Objects, Systems and Technologies.

[125] Wendler J., Maraite D., Nocke A., Cherif C. (2020). Novel textile moisture sensors based on multi-layered braiding constructions. Text. Res. J..

[126] Banitaba S.N., Ehrmann A. (2021). Application of electrospun nanofibers for fabrication of versatile and highly efficient electrochemical devices: A review. Polymers.

[127] Coyle S., Morris D., Lau K.-T., Diamond D., Taccini N., Costanzo D., Salvo P., di Francesco F., Trivella M.G., Porchet J.-A. Textile sensors to measure sweat pH and sweat-rate during exercise. Proceedings of the 2009 3rd International Conference on Pervasive Computing Technologies for Healthcare.

[128] Possanzini L., Decataldo F., Mariani F., Gualandi I., Tessarolo M., Scavetta E., Fraboni B. (2020). Textile sensors platform for the selective and simultaneous detection of chloride ion and pH in sweat. Sci. Rep..

[129] Promphet N., Rattanawaleedirojn P., Siralertmukul K., Soatthiyanon N., Potiyaraj P., Thanawattano C., Hinestroza J.P., Rodthongkum N. (2019). Non-invasive textile based colorimetric sensor for the simultaneous detection of sweat pH and lactate. Talanta.

[130] Caldara M., Colleoni C., Guido E., Re V., Rosace G. (2016). Optical monitoring of sweat pH by a textile fabric wearable sensor based on covalently bonded litmus- 3-glycidoxypropyltrimethoxysilane coating. Sens. Actuators B Chem..

[131] Morris D., Coyle S., Wu Y.Z., Lau K.T., Wallace G., Diamond D. (2009). Bio-sensing textile based patch with integrated optical detection system for sweat monitoring. Sens. Actuators B Chem..

[132] He J., Xiao G., Chen X.D., Qiao Y., Xu D., Lu Z.S. (2019). A thermoresponsive microfluidic system integrating a shape memory-modified textile and a paper-based colorimetric sensor for the detection of glucose in human sweat. RSC Adv..

[133] Siripongpreda T., Somchob B., Rodthongkum N., Hoven V.P. (2021). Bacterial cellulose-based re-swellable hydrogel: Facile preparation and it potential application as colorimetric sensor of sweat pH and glucose. Carbohydr. Polym..

[134] Zhou Y., Han T., Naw N.P.P., Lammy A.V., Goh C.H., Boujday S., Steele T.W.J. (2016). Real-time colorimetric hydration sensor for sport activities. Mater. Des..

[135] Venkatesan M., Veeramuthu L., Liang F.-C., Chen W.-C., Cho C.-J., Chen C.-W., Chen J.-Y., Yan Y., Chang S.-H., Kuo C.-C. (2020). Evolution of electrospun nanofibers fluorescent and colorimetric sensors for environmental toxicants, pH, temperature, and cancer cells–a review with insights on applications. Chem. Eng. J..

[136] Gualandi I., Tessarolo M., Mariani F., Possanzini L., Scavetta E., Fraboni B. (2021). Textile chemical sensors based on conductive polymers for the analysis of sweat. Polymers.

[137] Terse-Thakoor T., Punjiya M., Matharu Z., Lyu B., Ahmad M., Giles G.E., Owyeung R., Alaimo F., Baghini M.S., Brunyé T.T. (2020). Thread-based multiplexed sensor patch for real-time sweat monitoring. Npj Flex. Electron..

[138] Coppedè N., Giannetto M., Villani M., Lucchini V., Battista E., Careri M., Zappettini A. (2020). Ion selective textile organic electrochemical transistor for wearable sweat monitoring. Org. Electron..

[139] Gualandi I., Marzocchi M., Achilli A., Cavedale D., Bonfiglio A., Fraboni B. (2016). Textile organic electrochemical transistors as a platform for wearable biosensors. Sci. Rep..

[140] Jose M., Oudebrouckx G., Bormans S., Veske P., Thoelen R., Deferme W. (2021). Monitoring body fluids in textiles: Combining impedance and thermal principles in a printed, wearable, and washable sensor. ACS Sens..

[141] Hexoskin. https://www.hexoskin.com/.

[142] Ambiotex. https://www.ambiotex.com/.

